# Single-tube collection and nucleic acid analysis of clinical samples for SARS-CoV-2 saliva testing

**DOI:** 10.1038/s41598-022-07871-4

**Published:** 2022-03-10

**Authors:** Kyle H. Cole, Alexis Bouin, Caila Ruiz, Bert L. Semler, Matthew A. Inlay, Andrej Lupták

**Affiliations:** 1 Department of Molecular Biology and Biochemistry, University of California, Irvine, Irvine, CA 92697 USA; 2 Department of Microbiology and Molecular Genetics, University of California, Irvine, Irvine, CA 92697 USA; 3 Department of Pharmaceutical Sciences, University of California, Irvine, Irvine, CA 92697 USA; 4 Sue and Bill Gross Stem Cell Research Center, University of California, Irvine, Irvine, CA 92697 USA; 5 Department of Chemistry, University of California, Irvine, Irvine, CA 92697 USA

**Keywords:** Viral infection, Analytical biochemistry, Public health, Laboratory techniques and procedures

## Abstract

The SARS-CoV-2 pandemic has brought to light the need for expedient diagnostic testing. Cost and availability of large-scale testing capacity has led to a lag in turnaround time and hindered contact tracing efforts, resulting in a further spread of SARS-CoV-2. To increase the speed and frequency of testing, we developed a cost-effective single-tube approach for collection, denaturation, and analysis of clinical samples. The approach utilizes 1 µL microbiological inoculation loops to collect saliva, sodium dodecyl sulfate (SDS) to inactivate and release viral genomic RNA, and a diagnostic reaction mix containing polysorbate 80 (Tween 80). In the same tube, the SDS-denatured clinical samples are introduced to the mixtures containing all components for nucleic acids detection and Tween 80 micelles to absorb the SDS and allow enzymatic reactions to proceed, obviating the need for further handling of the samples. The samples can be collected by the tested individuals, further decreasing the need for trained personnel to administer the test. We validated this single-tube sample-to-assay method with reverse transcription quantitative real-time polymerase chain reaction (RT-qPCR) and reverse transcription loop-mediated isothermal amplification (RT-LAMP) and discovered little-to-no difference between Tween- and SDS-containing reaction mixtures, compared to control reactions. This approach reduces the logistical burden of traditional large-scale testing and provides a method of deployable point-of-care diagnostics to increase testing frequency.

## Introduction

The pandemic caused by SARS-CoV-2 (severe acute respiratory syndrome coronavirus 2)^1^ is an ongoing health crisis that has put a spotlight on current methodologies of viral detection. Large scale testing has become a part of the weekly routine to help quell viral spread. In the early stages of the global pandemic, SARS-CoV-2 testing was mainly reserved for those who were symptomatic or had been exposed to other virus-positive individuals, limiting test availability for asymptomatic or pre-symptomatic individuals. Bottlenecks in testing assisted in the spread of SARS-CoV-2 because asymptomatic individuals make up a large portion of the SARS-CoV-2 infected population^2^. While testing has expanded in recent months to include asymptomatic individuals, the length of time between sample collection and result determination has hindered our ability to identify positive individuals before they become infectious and capable of spreading the virus. This lag in test turnaround is due in part to the lengthy sample processing, RNA purification step, and diagnostic assay setup. Furthermore, the high demand for sterile swabs, buffers, tubes, pipette tips and PCR reagents has led to supply chain complications that have further impeded efforts to expand testing capability. Lastly, the number of steps required—from sample collection to result notification—has placed tremendous pressure on clinical labs and staff to avoid mix-ups that could lead to incorrect diagnoses.

Safety is also an important issue, because specimens are potentially infectious during transport until inactivated during processing. While newer methods involving saliva collection have eliminated many of the RNA purification steps, several processing steps remain, and these methods have not had the transformative impact on testing as envisioned. At-home testing solutions, while promising, are still in development and are expensive on a per-test basis. These issues highlight the need for improved testing methods. Current diagnostic-testing workflows for nucleic-acid based methods require long processing times, hindering the time between testing and result determination. Lag in test turnaround is in part due to the RNA purification process necessary to achieve optimal test sensitivity, which has been weighted as the diagnostic standard^3^. While sensitivity of each test is important, testing frequency is also a critical factor for early detection in households and communities, necessitating the development of simple, fast, and on-site testing technologies^3,4^.

Standard methods of viral diagnostics rely on viral transport media (VTM) to preserve the specimen after collection from the patient. VTM does not inactivate viruses; therefore, patient specimens collected in VTM are potentially infectious. While inactivation is performed by sample heating or by the addition of sodium dodecyl sulfate (SDS)^5^, these steps are performed after transportation and delivery to a diagnostic facility. Recent methods have aimed to streamline this process of viral inactivation and genomic RNA extraction for RT-qPCR^6,7^, RT-LAMP^8–10^, and CRISPR/Cas9^11–13^ by utilizing a heat inactivation step prior to assessment. However, these techniques require further handling of the inactivated samples, such as transfer of specific volumes of the sample material into the diagnostic solution for downstream analysis, thus prolonging the process and requiring dedicated personnel or robotic facilities to handle the samples.

We sought to expedite the inactivation of viruses, bacterium, and human cells by employing the common anionic surfactant, dodecyl sulfate (SDS), for the denaturation of viral and cellular structures and subsequent release of RNA with the addition of ethylenediaminetetraacetic acid (EDTA) to chelate divalent metal ions required for nuclease activity (Tris buffer is used to maintain a near neutral pH). Others have utilized similar approaches using surfactants (SDS, polysorbate [Tween], Triton X-100, and nonyl phenoxypolyethoxylethanol [NP-40])^5^, reducing agents (e.g., tris(2-carboxyethyl)phosphine [TCEP], dithiothreitol [DTT] to inactivate RNase A family of enzymes) and EDTA, and/or heat inactivation^8,14^. While the inclusion of SDS at low millimolar concentrations into any reaction mixture will inactivate common pathogenic viruses, it also leads to protein denaturation^15^ and is thus incompatible with enzymatic activity, such as subsequent RT and PCR steps. However, SDS can be sequestered by non-ionic surfactants, such as Tween, at or above their critical micelle concentration (CMC) via hydrophobic interactions^16^. The inclusion of Tween in an enzymatic reaction mixture therefore abrogates the inhibitory effects of SDS through adsorption by the Tween micelles, allowing for SDS-treated clinical samples to be added directly to an enzyme reaction mixture. Once adsorbed into the Tween micelles, SDS does not denture any proteins in the reaction mixture, allowing for workup of the RNAs (and DNAs) present in the sample using enzymes.

**Figure 1:**
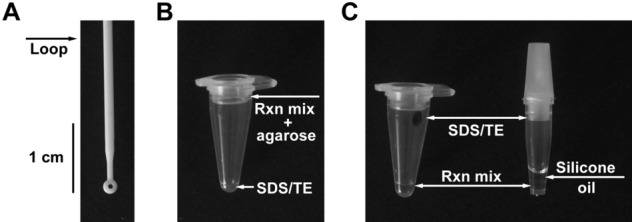
Inoculation loop for sample acquisition and configuration of reaction tubes. (**A**) A 1 µL inoculation loop is used for oral sample withdrawal and collection. (**B**) A 0.1 mL PCR tube with a Tween-containing reaction mixture immobilized by agarose is placed inside the cap of the tube and 1 µL of SDS/TE solution is placed at the bottom. A clinical sample withdrawn using the inoculation loop is transferred to the bottom of the tube and denatured by mixing it with the SDS/TE solution using the same loop. Subsequently, the nucleic acid detection mixture Tween 80 is transferred from the cap to the bottom of the tube by centrifugation or shaking. (**C**) Alternatively, 0.1 mL PCR tube with a dried 1 µL spot of SDS/TE on the sidewall of the tube and 5 µL of a Tween-containing reaction mixture at the bottom can be used (left tube). The inoculation loop is rubbed on the side of the tube to dissolve the SDS/TE mixture (marked with permanent marker) and denature the clinical sample and then used to drive the sample to the bottom of the tube. For systems with optical readout at the bottom of the sample tube (e.g., Biomolecular Systems Mic, Quantabio Q tube or Qiagen RotorGene Q) the SDS/TE solution can be dried on the sidewall and the Tween-containing RT-qPCR reaction mixture can be stored underneath 5 µL of oil (right tube).

Current clinical sample procurement methods are accomplished using sterile swabs for the collection of nasopharyngeal or nasal samples, in addition to saliva collection, by utilizing specially fitted collection tubes for direct oral deposition. We sought a simple-to-use method of sample acquisition that could be easily mastered by untrained individuals for self-collection. Plastic inoculation loops used in microbiology filled this need, because they are inexpensive, disposable, and can reproducibly remove specific volumes (e.g., 1 µL) of saliva (Fig. 1A). These loops provide a means of consistent, small-volume saliva retrieval in addition to their convenient size for introduction into a PCR test tube. Viral samples retrieved by loop can be directly inactivated by applying the sample directly to an SDS/TE mixture and briefly mixing.

To utilize this system within a single tube, the SDS/TE solution must be initially separated from the enzyme reaction mixture. This was accomplished by immobilizing the reaction mixture with agarose in the lid of the tube, while supplying the SDS/TE mixture at the bottom (Fig. 1B). Alternatively, the SDS/TE could be heat-dried on the inner-side wall of the PCR tube with the reaction mix contained at the bottom of the PCR tube (Fig. 1C). This configuration allows for streamlined sample retrieval, inactivation by SDS/TE, and subsequent introduction into the Tween-containing enzymatic reaction mixture. Utilizing this approach, we demonstrate both RT-qPCR and RT-LAMP analyses of viral particles harboring the SARS-CoV-2 spike protein. The method thus represents a practical sample-to-assay approach with the potential to accelerate the diagnostic process as well as providing a cost-effective solution for increasing testing frequency.

## Results

### Optimization of SDS and Tween for viral inactivation and qPCR

**Figure 2:**
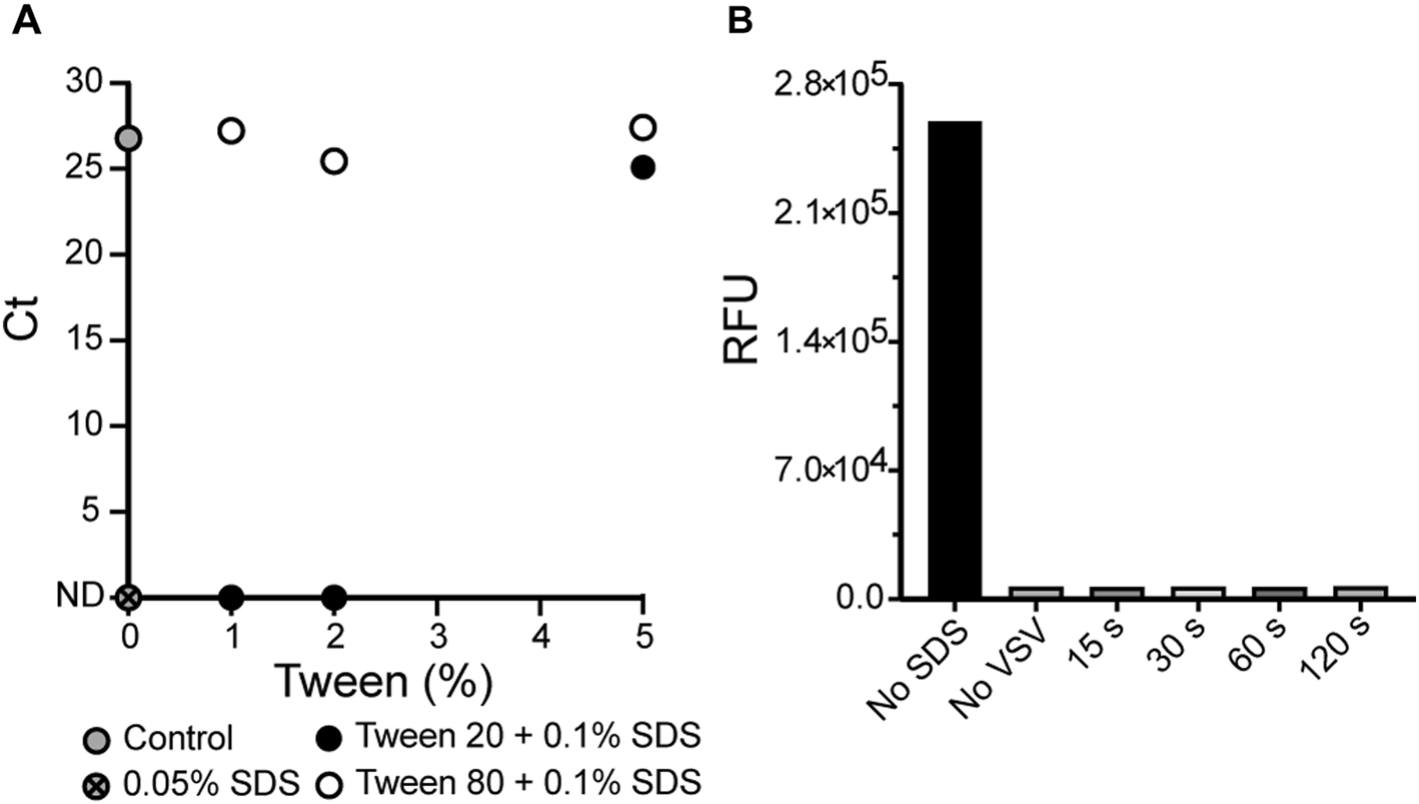
Effective SDS and Tween concentration determination for qPCR and virus inactivation. (**A**) Determination of the minimal Tween 20 and Tween 80 concentration necessary for SDS sequestration to enable amplification of 20,000 copies/µL of RNase P DNA. *Ct* cycle threshold, *ND* not detected. (**B**) Fluorescence assay of eGFP-expressing recombinant VSV-SARS-CoV-2 (10^7^ pfu/mL) infecting Vero-E6 cells after incubation with 0.1% (w/v) SDS for 15–120 s. Only a sample that was not exposed to SDS showed infectivity. 15 s exposure to 0.1% (w/v) SDS proved sufficient to completely inactivate the virus, likely due to dissolution of its membrane and denaturation of the sample proteins.

We first tested qPCR reactions to determine the concentration of Tween necessary to sequester SDS and prevent the inhibition of amplification. To test this, human RNase P gene (RPP30) was amplified from a control plasmid at varying concentrations of Tween 20 and Tween 80. We first established that 0.005% (w/v) final concentration of SDS is sufficient to inhibit the amplification reaction. We then increased the SDS concentration to 0.01% (w/v) and tested Tween 20 and Tween 80 at 1%, 2%, and 5% (v/v) for the ability to amplify DNA (Fig. 2A). At 5% (v/v) Tween 20 supported amplification in the presence of SDS but was ineffective at 1% and 2% (v/v). Tween 80 abolished the inhibitory effects of SDS at all three concentrations and supported amplification comparable to the control reaction. The results are consistent with the higher stability of the Tween 80 micelles, compared to Tween 20 (Tween 20 has a higher critical micelle concentration than Tween 80)^16,17^. While not directly tested, we chose to use 3% (v/v) Tween 80 for our subsequent applications as it was easier to consistently pipette in comparison to the more viscous 5% (v/v) Tween 80 solutions.

A vesicular stomatitis virus (VSV) expressing enhanced green fluorescent protein (eGFP) and the SARS-CoV-2 spike protein (VSV-eGFP-SARS-CoV-2^18^), was used as a model system to test the SDS inactivation efficacy. Like SARS-CoV-2, VSV is an enveloped RNA virus that loses infectivity when the envelope is disrupted by detergent treatment. To assess virus inhibition, 1 µL inoculation loops were dipped into a VSV-eGFP-SARS-CoV-2 viral suspension of 10^7^ pfu/mL and introduced to a heat dried 1 µL spot containing 0.1% (w/v) SDS in a Tris–EDTA buffer on the inner-side wall of a PCR tube. The dried spot was indicated with a permanent marker to better identify the location of the SDS/TE. The 1 µL viral samples on the inoculation loops were mixed on the SDS spot for 15–120 s prior to introduction into a 5 µL RT-qPCR reaction mixture containing 3% (v/v) Tween 80 at the bottom of the PCR tube. To prevent cross contamination, a new 1 µL loop was used to remove 1 µL from the RT-qPCR reaction mixtures and inoculate Vero-E6 cell monolayers grown 24 h prior to infection. Cells were grown for 5 days post-infection with daily fluorescent signal monitoring and until the untreated viral sample achieved complete infection. After incubation, eGFP expression in SDS-treated, no-SDS, and no-VSV samples was measured by fluorescence. Inhibition of VSV-eGFP-SARS-CoV-2 was observed at the minimum time of mixing on the 0.1% (w/v) SDS/TE spot at 15 s and was comparable to mixing for longer periods and to the no-VSV control. Greater eGFP expression was observed in the untreated VSV-eGFP-SARS-CoV-2 control. The substantial fluorescence observed in the untreated sample demonstrates that SDS fully inactivates the virus, but the 3% (v/v) Tween 80 dissolved in the enzyme reaction mixture does not. These results illustrate that 0.1% (w/v) SDS with 15 s of mixing is sufficient to inactivate VSV-eGFP-SARS-CoV-2.

### Comparison of immobilization methods

**Figure 3:**
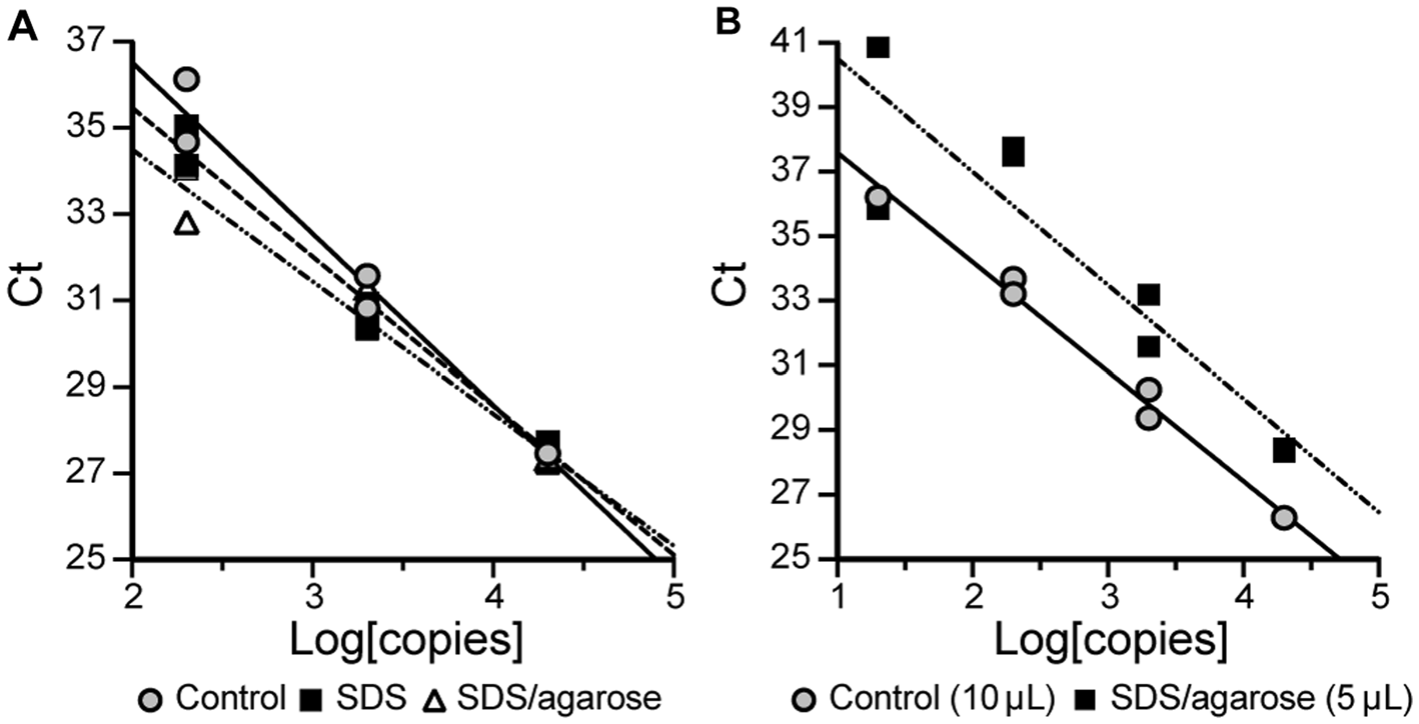
Comparison of standard qPCR to SDS/Tween and SDS/Tween agarose reactions. (**A**) Comparison of 10 µL qPCR reactions for SARS-CoV-2 N1 gene DNA amplification, containing 3% (v/v) of Tween 80 with (R^2^ = 0.9781) and without 0.01% SDS (w/v, final; R^2^ = 0.9818) and 1% (w/v) agarose (R^2^ = 0.9260). (**B**) Comparison of a 10 µL qPCR reaction for SARS-CoV-2 N1 gene DNA containing 3% (v/v) Tween 80 (R^2^ = 0.9908) with a 5 µL qPCR reaction (R^2^ = 0.8451) for SARS-CoV-2 N1 DNA detection, containing 3% (v/v) Tween 80, 0.01% SDS (w/v, final) and 1% (w/v) agarose. In all conditions containing SDS, the DNA samples were initially incubated with 0.1% SDS (w/v) in Tris/EDTA buffer to simulate denaturing conditions for clinical samples. *Ct* cycle threshold.

For point-of-care testing, immobilization of the master mix is necessary to prevent the mixing of the Tween-80-containing enzyme/primer master reaction with the SDS prior to virus denaturation, particularly when SDS is supplied at the bottom of the reaction tube. Several methods can be used for the separation of the reagents within a single tube: (1) mineral oil or silicone oil can be overlaid above the enzyme mix^19^ (Fig. 1C), (2) low-melting temperature wax can be overlaid on top of the mixture and solidified to make a solid barrier at room temperature^20^, or (3) low-melting-point agarose can be introduced into the mixture at a slightly elevated temperature (e.g., 42 °C) and then stored at room temperature or lower to immobilize the mixture. We confirmed that both the oil overlay and the agarose inclusion supported immobilization of the enzyme mixture, and for most experiments, we used low-melting-point agarose for its ability to melt at the low reaction temperatures (≤ 50 °C), enzyme stabilization^21^, and its optical transparency^22^. The addition of 1% (w/v) agarose in the presence of SDS (0.01% (w/v) final) and 3% (v/v) Tween 80 showed no discernable inhibition of the amplification of SARS-CoV-2N gene from a SARS-CoV-2 plasmid when compared to reactions containing 3% (v/v) Tween 80, with and without 0.01% SDS (w/v, final) in 10 µL samples (Fig. 3A).

Next, we compared a 5 µL reaction containing the SDS/Tween/agarose to a standard 10-µL reaction mixture containing 3% (v/v) Tween 80 to test any inhibitory effects under more stringent conditions (Fig. 3B). The smaller reaction volume with the addition of both 0.01% SDS (w/v, final) and 1% (w/v) agarose increased the number of cycles necessary for detection of SARS-CoV-2 N1 at each concentration of plasmid DNA, thus exhibiting a slight decrease in amplification efficiency (Fig. 3B). Importantly, a 5 µL reaction with a sample introduced into a 1 µL drop of SDS solution and subsequently mixed with a master mix containing Tween 80 allowed quantitative detection of DNA plasmids.

### RT-qPCR of SARS-CoV-2 patient RNA and viral particles

**Figure 4:**
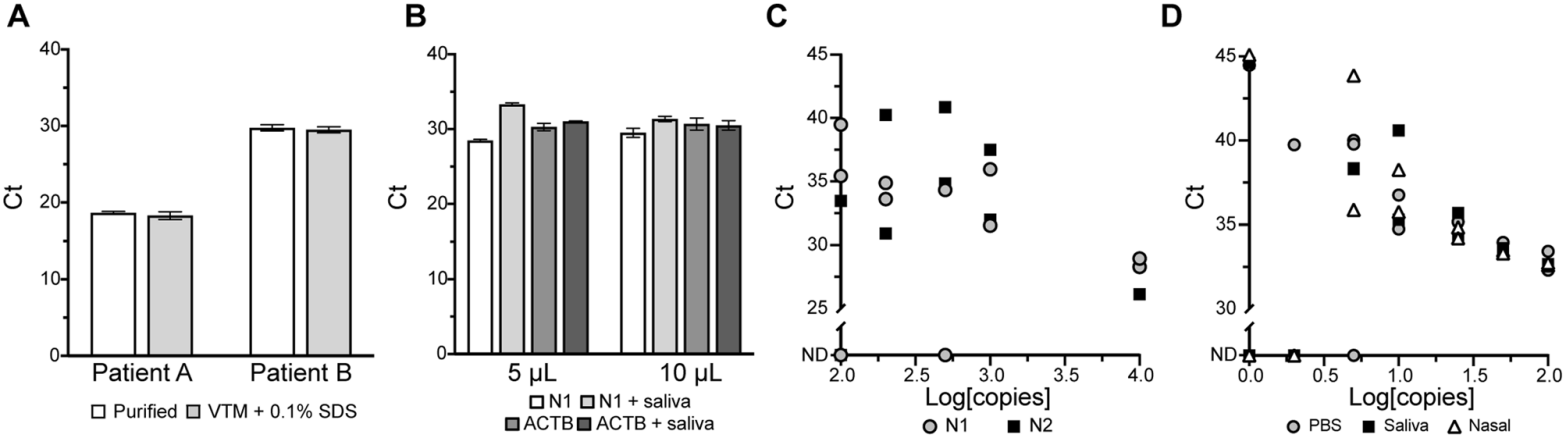
Single-tube sample-to-assay RT-qPCR of SARS-CoV-2 positive-patient RNA sample and inactivated viral particles. (**A**) Comparison of five-fold diluted purified and unpurified SARS-CoV-2 patient RNA samples (nasal swabs stored in VTM + 0.5% [w/v] SDS) in triplicate 10 µL RT-qPCR reactions containing SARS-CoV-2 N1 primers and probe, 3% (v/v) Tween 80, and 0.5% (w/v) agarose. Samples were diluted five-fold to achieve a 0.1% (w/v) SDS working concentration for unpurified samples in VTM. Data are presented as ± SEM. (**B**) Comparison of 5 µL and 10 µL RT-qPCR reactions containing 3% (v/v) Tween 80, and 0.5% (w/v) agarose with a purified SARS-CoV-2 patient RNA sample. Triplicate reactions were performed at each volume, comparing the patient sample with and without spiking into saliva. Reactions were performed with SARS-CoV-2 N1 and beta-actin (ACTB) primer–probe sets. Data are presented as ± SEM. (**C**) RT-qPCR results of SARS-CoV-2 synthetic RNA (n = 11) in water. RT-qPCR was performed for 45 cycles using SARS-CoV-2 N1- and N2-specific primers and probes on a Quantabio Q thermal cycler. Samples were tested blind with concentrations and storage media revealed thereafter (ND = not detected). (**D**) Scatter plot of Ct value (y-axis) versus log[copies] of SARS-CoV-2 viral particles (x-axis) of inactivated SARS-CoV-2 viral particle samples (n = 49) in PBS, saliva, and nasal media. RT-qPCR was performed for 45 cycles with SARS-CoV-2 N1-specific primers and fluorescent probe on a Bio-Rad CFX Connect thermal cycler. *Ct* cycle threshold.

While the SDS/Tween assay was compatible with qPCR, understanding RT-qPCR functionality was necessary to establish a method for sample-to-assay assessment of infection. To assess RT-qPCR, 50 µL of two positive SARS-CoV-2 patient nasal swabs samples stored in VTM with 0.5% (w/v) SDS were purified using a Zymo Research Quick DNA/RNA-viral extraction kit and eluted in an equivalent volume of nuclease-free water. We diluted the unpurified RNA (VTM with 0.5% [w/v] SDS) fivefold in nuclease-free water to reach an effective SDS concentration of 0.1% (w/v) and compared that to equally dilute purified samples (Fig. 4A). Reactions were performed at 10 µL with the RT-qPCR reaction mixture immobilized in the lid of the reaction tube (0.1 mL optically transparent qPCR tubes). The enzyme mixture also contained SARS-CoV-2 N1 primers and probe, 3% (v/v) Tween 80, and 0.5% agarose (w/v). Samples were prepared in triplicate with 1 µL of sample added by pipetting. Amplification of both purified and unpurified patient samples provided consistent Ct values with no observable differences between them.

We then assessed one purified patient RNA sample that was spiked into saliva obtained from one of our authors to determine whether saliva could inhibit or interfere with detection (Fig. 4B). Reactions were performed at two volumes (5 µL and 10 µL) and contained SARS-CoV-2 N1 and beta-actin (ACTB) primer–probe sets, 3% (v/v) Tween 80, and 0.5% agarose (w/v). Samples were prepared in triplicate, retrieved with 1-µL inoculation loops, and inactivated by mixing with 0.1% (w/v) SDS in TE and 5 mM DTT^8^ stored at the bottom of the reaction tube. Reactions were performed using the previously mentioned CDC-recommended protocol. A small difference in amplification of SARS-CoV-2 N1 was observed at both reaction volumes when the RNA sample was spiked into saliva. However, this same difference was not observed for ACTB between either the 5 µL or 10 µL reaction volumes. We hypothesize that this difference is due to the purified-patient RNA degrading when incubated in saliva, prior to inactivation by SDS. The ACTB mRNA was likely protected within cells collected with saliva and released after SDS treatment, explaining the consistency of the Ct values in the ACTB reactions with and without saliva. Overall, these results showed that the reaction mixture containing both Tween 80 and agarose can detect SDS treated SARS-CoV-2 RNA.

Next, we tested our sample-to-assay method utilizing an RT-qPCR reaction mixture overlaid with silicone oil. We tested samples of synthetic SARS-CoV-2 RNA stored in water (n = 11, provided by the XPRIZE Foundation; Fig. 4C). The RT-qPCR reaction mixture was prepared and stored beneath a layer of silicone oil in a Quantabio Q tube produced specifically for the Q qPCR instrument, which measures fluorescence from the bottom of the tubes by rotating the samples above immobile excitation LEDs and photomultiplier detectors. This process also serves to centrifuge the samples to the bottom of the tubes. We assessed these samples by first collecting each sample using an inoculation loop and mixing the sample on a dried SDS/TE spot on the side of the tube for 15 s, which was marked with a permanent marker. After mixing, the sample was driven by the inoculation loop to the bottom of the test tube (past the silicone oil) into the reaction mixture. Because the samples consisted of synthetic RNA stored in water, it was expected that some of the synthetic SARS-CoV-2 RNA had degraded. This experiment provides an example of this method when utilizing a system that reads fluorescence from the bottom of the tube and maintains partitioning of the reaction mixture with silicone oil. Additionally, this provides a useful method of detection of > 200 copies/µL of viral RNA.

We next assessed 49 inactivated SARS-CoV-2 viral particle samples diluted in phosphate buffered saline (PBS), saliva, and nasal media, provided as blind samples by the XPRIZE Foundation (Fig. 4D). Reaction tubes (0.1 mL optically transparent qPCR tubes) were prepared by drying 1 µL of 0.1% (w/v) SDS in TE buffer solution on the inner-side wall of the PCR tube. Samples were collected by dipping 1 µL inoculation loops in the stored samples, swirled on the dried SDS for 15 s and subsequently driven to the bottom of the tube containing a 5 µL RT-qPCR reaction mixture that included 3% (v/v) Tween 80 and N1-specific primers and probe. Reactions containing 100, 50, and 25 copies of SARS-CoV-2 viral particles appeared at similar cycles regardless of storage media (n = 18) while samples containing 10 copies varied between 35 and 40 cycles (n = 7) with greater variability and false-negative results at lower concentrations (Fig. 4D). Based on these results, we estimate our limit-of-detection (LOD) to be 10 copies/µL.

### Sample-to-assay RT-LAMP with the addition of Tween 80 and SDS

**Figure 5:**
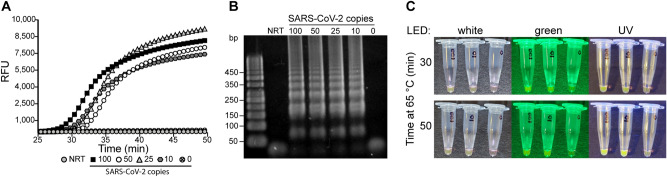
RT-LAMP amplification with SDS and Tween 80. (**A**) Real-time fluorescence of SARS-CoV-2N gene amplification by RT-LAMP was performed at 65 °C for 50 min. Indicated concentrations (copies) of SARS-CoV-2 viral particles were assessed including a no-reverse-transcriptase (NRT) and no-template control (NTC; 0 copies), with all SARS-CoV-2 samples providing a positive result after ~ 30 min. (**B**) Amplification products (1 µL) from the real-time fluorescence RT-LAMP reaction were fractionated using agarose gel electrophoresis. The expected laddering pattern of LAMP amplification products was observed at all SARS-CoV-2 viral particle concentrations and not observed in NRT and NTC controls. The uncropped gel can be found in Supplementary Fig. 1. (**C**) Fluorescence of RT-LAMP reactions of SARS-CoV-2 viral particles containing 20 µM SYTO 82 observed after 30 and 50 min of incubation at 65 °C. Reactions were analyzed with white, green, and UV (365 nm) handheld LED flashlights at each time point, with readily observable fluorescence of amplification reactions starting from 100 to 10 copies (labeled) after 30 min with each illumination. After 50 min, fluorescence in the 100-copy and 10-copy tubes increased with no observable amplification in the 0-copy control. *RFU* relative fluorescence units.

After verifying sensitive, reproducible RT-qPCR detection with SDS-denatured samples and stabilization of the enzymatic reactions with Tween, we then tested RT-LAMP for a single-tube sample collection and analysis. RT-LAMP has key advantages over RT-qPCR because it is performed isothermally and does not require additional equipment for readout, resulting in diagnostic simplicity. These advantages make RT-LAMP a promising tool for point-of-care detection of viral infection^8–10,23–27^. Previous work has established that RT-LAMP can withstand 3% (v/v) Tween 20 in addition to 3% (v/v) Triton X-100 with little inhibition of amplification^8^. To validate RT-LAMP detection of RNA samples in the presence of SDS and Tween, real-time fluorescence measurements were collected on a real-time PCR thermal cycler, with the addition of the nucleic-acid intercalating dye SYTO 82^28–30^. Four concentrations of SARS-CoV-2 viral particles (100, 50, 25, and 10 copies/µL) were collected using 1 µL inoculation loops and incubated at 65 ºC for 50 min in the RT-LAMP reaction. Amplification of 100 to 10 copies of RNA was detected after 33 min using N1-specific primers designed by Huang et al. (Fig. 5A)^31^. Non-specific amplification was not observed in no-reverse-transcriptase (NRT) and 0 copy controls, which was further confirmed by agarose gel electrophoresis (Fig. 5B).

As mentioned, the isothermal reaction of RT-LAMP eliminates the need for expensive detectors, and with additional indicators, positive results can be observed without costly equipment or gel electrophoresis. Previous methods have utilized pH (e.g., phenol red) or complexometric dyes (e.g., hydroxynaphthol blue), and nucleic acid intercalators (e.g., SYBR Green, EvaGreen, and SYTO 82) for the detection of target RNA^8,9,23,27,29,32,33^. After validating RT-LAMP using real-time fluorescence, we hypothesized that amplification can be observed without dedicated fluorescence readers. We tested this by increasing the concentration of SYTO 82 in the RT-LAMP reaction from 5 to 20 µM. Amplification of 100 copies and 10 copies of SARS-CoV-2 RNA was readily observed in 30 min at 65 °C, with no amplification observed in the NTC reaction after 50 min (Fig. 5C). These results were obtained under consumer-grade white, green, and UV (365 nm) excitation light using handheld LEDs and imaged by a mobile phone.

## Discussion

We present a method aimed at increasing the frequency of viral testing by decreasing the handling and time between sample collection and assessment for direct RNA detection in patient saliva within a single reaction tube (Fig. 1). In previous studies, nucleic acid detection techniques have required extraction of the genetic material from the clinical samples^8,11,12,31,34,35^, with some methods using heat denaturation to inactivate the pathogens and human cells in the clinical samples and release the genetic material for subsequent analysis^6–9,13,26,36,37^. In most cases, the sample collection and inactivation or extraction is performed in separate tubes from the analysis step and thus require additional handling by trained personnel. We sought to simplify the sample preparation to decrease the time and effort necessary to collect, denature, and analyze the sample. In effect, we have eliminated all pipetting and open-tube processing steps. If an individual collects their own specimen utilizing an inoculation loop (Fig. 1), adds it into the tube, and caps it, trained staff will only need to place the tube in the RT-qPCR instrument and analyze the results. After sample collection, the tube can be washed on the outside with a denaturing solution (e.g., soap or sanitizer) and placed in a tube rack or directly into the qPCR instrument by the tested individual, further eliminating handling by technical personnel.

To render the clinical samples noninfectious, we used laboratory detergent (SDS), which is commonly used to denature proteins and disintegrate lipid membranes. Detergent is the most active and widely available denaturant and, as expected, we observed that incubating a recombinant VSV-eGFP-SARS-CoV-2 virus sample for 15 s made the sample completely non-infectious (Fig. 2B). However, SDS also denatures enzymes that are used for nucleic acid detection, and to avoid a purification step, we required a mechanism of SDS sequestration from the solution. One approach is to precipitate the dodecyl sulfate with potassium, which, unlike the sodium salt, is insoluble; however, precipitation would lead to flocculates that could potentially hamper the downstream analysis of the solution. Instead, we chose to adsorb dodecyl sulfate into micelles formed by very stable surfactants, such as the Triton and Tween series of non-ionic detergents. Of the commercially available non-ionic detergents, Tween 80 forms the most stable micelles with low micromolar critical micelle concentration over a wide range of temperatures. Both Tween and Triton surfactants have been widely used in enzymatic reactions, including PCR and droplet PCR, and are therefore highly suitable for SDS sequestration. With the addition of Tween to an RT-qPCR reaction, the denaturing effects of the SDS are abolished, enabling amplification of target DNA (Fig. 2A).

The addition of agarose was tested to determine whether a stabilizing medium could be supplemented to better immobilize the reaction mixture away from the place where a small volume of SDS/TE was applied (and sometimes dried). We propose this approach as a means of immobilizing the reaction mixture during sample collection to prevent the loss of the reagents crucial for diagnostics. We observed no inhibition of qPCR due to SDS/Tween and agarose at equal reaction volumes (10 µL), when compared with standard reaction conditions (Fig. 3B), and the amplification efficiency was only slightly lower when 1 µL samples were incubated in 5 µL enzyme reactions. A similar result was observed for RT-qPCR utilizing a column-purified SARS-CoV-2 patient sample, even in the presence of saliva (Fig. 4B). We believe that this slight decrease in amplification efficiency is acceptable because the mean cycle threshold of N1 primer sets used in standard RT-qPCR protocols for self-saliva collection is 32.8 for asymptomatic patients and those with early- and late-onset of disease^38^.

Testing inactivated SARS-CoV-2 viral particles in PBS, saliva, and nasal media revealed variation at concentrations of 10 copies and below, giving us an estimated limit of detection of 10 copies/µL (Fig. 4D). Because others have illustrated a detection limit of 5.6 copies/µL of SARS-CoV-2 following the Centers for Disease Control and Prevention’s RT-qPCR guidelines for the N1-primer set, we believe our protocol falls within a meaningful detection range for this coronavirus^39^. This slight sensitivity decrease is compensated by the great reduction in diagnostic handling and turnaround time, thereby allowing for more frequent testing. Furthermore, 10 copies/µL is almost two orders of magnitude below the median viral load of sputum samples (752 copies/µL) collected from infected individuals^40^.

RT-LAMP has the potential to fill a niche previously occupied by rapid-antigen tests. It has been reported that rapid-antigen tests have a high false-negative rate in saliva samples with Ct ≥ 25 while RT-LAMP had greater sensitivity for samples with Ct ≤ 35^38^. Our method applied to RT-LAMP allowed for the detection of 10 copies (mean Ct = 34.8) of SARS-CoV-2 N1 RNA in 30 min (Fig. 5). By increasing the concentration of SYTO 82 in the RT-LAMP reaction, a positive result could be seen under white light and better observed with green and UV (365 nm) LEDs. Based on these results, RT-LAMP would require the same incubation time as commercial rapid-antigen tests (30 min) with greater sensitivity, although requiring incubation at 65 °C. Additionally, our method of RT-LAMP does not require the addition of buffering reagents typically required of lateral-flow rapid-antigen tests. While it does not have the same accuracy of RT-qPCR and suffers from non-specific amplification activity, RT-LAMP paired with this sample-to-assay method could replace the market of rapid-antigen tests while maintaining the simplicity of result determination.

## Methods

### Fluorescent VSV inactivation assay

Vero-E6 cells were grown in Dulbecco’s Modified Eagle Medium (DMEM) supplemented with 10% fetal bovine serum and a 1× antibiotic–antimycotic solution and maintained at 37 °C with 5% CO_2_. Vero-E6 cells were seeded 24 h prior to infection in 6-well plates. Infection was performed using VSV-eGFP-SARS-CoV-2 (generously provided by Paul W. Rothlauf and Sean P.J. Whelan at Washington University in St. Louis^18^) expressing SARS-CoV-2 spike protein (*S* gene). Following infection, cells were grown at 34 °C with 5% CO_2_ in DMEM supplemented with 10% FBS.

SDS inactivation was performed by dipping a 1 µL inoculation loop (Globe Scientific, 2810) in a VSV-eGFP-SARS-CoV-2 suspension at 10^7^ pfu/mL and applying the inoculation loop to a dried 1 µL spot of SDS/TE (0.1% w/v SDS, Tris–HCl, pH 8.0) on the side wall of a PCR reaction tube, followed by mixing for 15–120 s. The treated inoculation loop was then driven down into a RT-qPCR reaction mixture (see standard RT-qPCR methods) containing 3% (v/v) Tween 80 and disposed of. A new 1 µL inoculation loop was then used to sample the inoculated RT-qPCR reaction mixture and introduced into the culture media of Vero-E6 cells seeded in a 6-well culture plate. Cells were grown at 34 °C with 5% CO_2_ in DMEM supplemented with 10% FBS for 5 days. Cells were fixed in PBS supplemented by 3.7% formaldehyde for 20 min at room temperature and washed 3 times with phosphate buffered saline (PBS). Fluorescence was measured using a CLARIOstar Plus Microplate Reader.

### DNA and RNA controls

Human RNase P gene (*RPP30*) was amplified from the Hs_RPP30_Positive control plasmid (Integrated DNA Technologies, IDT, 10006626). SARS-CoV-2 N1 DNA was amplified from the 2019-nCoV_N_Positive control plasmid (IDT, 10006625). SARS-CoV-2 viral particles and synthetic RNA were provided by the XPRIZE Foundation (Team ID: 3650) and produced by ZeptoMetrix and Twist Biosciences, respectively. Viral particles were stored at 4 °C per manufacturer recommendation. Synthetic RNA was stored at − 80 °C per manufacturer recommendation.

### Purification of SARS-CoV-2 patient samples

Human specimen samples were obtained under an institutional review board (IRB) approved protocol (HS# 2012-8716) at the University of California, Irvine (UCI). Specimens collected originally for diagnostic purposes were processed and stored in the University of California, Irvine Medical Center hospital. Samples were collected under the HS# 2012-8716 IRB approved protocol as post-diagnostic remnants from the Pathology Department at the University of California, Irvine, and processed for research use by the Chao Family Comprehensive Cancer Center Experimental Tissue Shared Resource. All experimental protocols were approved by the UCI IRB and the UCI Institutional Biosafety Committee (IBC). UCI IRB categorized our protocol as Non-Human Subject Research, since de-identified specimens, stripped of all personal health information (PHI) were received from the Experimental Tissue Resource (HS# 2012-8716) and the UCI IBC (BUA-R191) covered the usage of inactivated, denatured specimens.

Pharyngeal swabs were maintained at 4 °C until initial diagnostic test was performed, followed by freezing in the original collection tubes at − 80 °C. At the time of releasing for research, the swabs were thawed and aliquoted at desired volumes, inactivated by incubation at room temperature for 30 min in 0.5% SDS^5^, and released to the investigators. A 50 µL volume of inactivated samples, in viral transport media (VTM) and 0.5% (w/v) SDS, were purified using the Zymo Research Quick-DNA/RNA viral extraction kit (D7020) following the manufacturer’s protocol and eluted in 50 µL of nuclease-free water.

### Standard RT-qPCR reactions

Standard RT-qPCR reactions were performed using either 2× qScript XLT 1-Step RT-qPCR ToughMix (Quantabio) or 4× TaqPath 1-Step Multiplex Master Mix (Invitrogen) at 1× final concentration. Forward and reverse primers (Supplementary Table 1) were used at 500 nM with hydrolysis probes at 125 nM final concentration. Reactions were performed at either 5 µL or 10 µL after the addition of dH_2_O to a desired volume and 1 µL of DNA/RNA samples. Reactions were measured on a Bio-Rad CFX Connect for 25 °C for 2 min, 50 °C for 15 min, 95 °C for 2 min, and 45 cycles of 95 °C for 3 s, and 55 °C for 30 s.

### RT-qPCR with SDS, Tween, and with/without agarose

RT-qPCR reactions containing SDS and Tween 80 were assembled similarly to the standard RT-qPCR reaction mixtures and contained 1× RT-qPCR master mix, 500 nM forward and reverse primers, and 125 nM of hydrolysis probes. Tween 80 was added to a desired final concentration of 3% (v/v). The SDS solution (0.1% [w/v] SDS in 10 mM Tris–HCl and 0.1 mM EDTA, pH 8.0) was added at a volume of 1 µL to the bottom of a 0.1 mL flat-top optically transparent PCR tube (Thomas Scientific) or added to the inner-side wall of the tube and allowed to dry at 70 °C for 5–10 min. The SDS spot was marked with permanent marker to identify its location.

For agarose containing reactions, ultra-low gelling temperature agarose (Sigma Aldrich, A2576) was prepared at a working concentration of 2.5–5% (w/v) in dH_2_O by heating the mixture to 80 °C for 2 min and holding at 42 °C. Warm agarose was added to a RT-qPCR reaction mix (1× RT-qPCR master mix, 500 nM of forward and reverse primers, 125 nM hydrolysis probes, and 3% [v/v] Tween 80) to a final concentration of 0.5–1% (w/v). The mixture was then added to the lid or to the bottom of a 0.1 mL optically clear flat-top PCR tube and allowed to solidify at room temperature.

For oil overlayed reactions, RT-qPCR reaction mixtures were added beneath the 5 µL silicone oil layer in a Q Tube (Quantabio, 95910-20). The SDS solution (0.1% [w/v] SDS in 10 mM Tris–HCl and 0.1 mM EDTA, pH 8.0) was added at a volume of 1 µL to the side of the tube and allowed to dry. Samples were added via 1 µL inoculation loop directly to the SDS spot and mixed for 15 s prior to being driven down into the silicone oil/reaction mixture at the bottom of the tube. Samples were incubated on a Quantabio Q thermal cycler for 37 °C for 30 s, 42 °C for 4 min, 50 °C for 5 min, 95 °C for 1 min, and 45 cycles of 95 °C for 5 s, and 55 °C for 10 s.

### RT-LAMP

5× RT-LAMP reaction buffer was prepared using 10× Isothermal Amplification Buffer (New England Biolabs; NEB) with the addition of 40 mM MgSO_4_, 25 µM SYTO 82 (Invitrogen, S11363), 4.5% (v/v) Tween 80 and dH_2_O to a preferred volume. RT-LAMP reactions contained 2 µL 5× RT-LAMP reaction buffer (1× reaction buffer: 20 mM Tris–HCl, 10 mM (NH_4_)_2_SO_4_, 50 mM KCl, 10 mM MgSO_4_, 0.1% [v/v] Tween 20, 0.9% [v/v] Tween 80, 5 µM SYTO 82, pH 8.8), 2 µL 7 mM dNTPs, 1 µL *Bst* 2.0 WarmStart DNA polymerase (8 U/µL) (NEB) 0.25 µL WarmStart RTx reverse transcriptase (150 U/µL) (NEB), and 1 µL 10× SARS-CoV-2 N1 LAMP primer mix (2 µM F3/B3, 16 µM FIP/BIP, 4 µM LF/LB; Supplementary Table 1) and dH_2_O to 9 µL. Prior to the addition of the reaction mixture, 1 µL 0.1% (w/v) SDS in 10 mM Tris–HCl pH 8, and 0.1 mM EDTA was added to the bottom of a 0.1 mL flat-top optically transparent PCR tube. Samples were collected with 1 µL inoculation loops and mixed in the SDS solution for 15 s. After sample addition, the reaction mixture was added for a final volume of 10 µL. Reactions were monitored using a Bio-Rad CFX Connect thermal cycler at 65 °C for 50 min.

End-point assays were prepared under identical conditions to real-time RT-LAMP reactions with a final concentration of 20 µM SYTO 82 for improved visualization. Reactions were incubated for 30 min and 50 min and visualized by white, green and UV (365 nm) LED lights. Images were taken by mobile phone.

## Supplementary Information

Supplementary Information.
