# Supplementary material for: Single-tube collection and nucleic acid analysis of clinical samples for SARS-CoV-2 saliva testing
Source: Sci Rep. 2022 Mar 10;12:3951. doi: 10.1038/s41598-022-07871-4 (PMC8913774; doi:10.1038/s41598-022-07871-4)
Supplement: Supplementary file 1 — Supplementary Information. [file 41598_2022_7871_MOESM1_ESM.pdf]

Single-tube collection and nucleic acid analysis of clinical samples for SARS-CoV-2 saliva testing

Kyle H. Cole[1], Alexis Bouin[2], Caila Ruiz[3], Bert L. Semler[2], Matthew A. Inlay[1,4], Andrej Lupták[1,3,5,*]

[1]Department of Molecular Biology and Biochemistry, University of California, Irvine, Irvine, California 92697, United States
[2]Department of Microbiology and Molecular Genetics, University of California, Irvine, Irvine, California 92697, United States
[3]Department of Pharmaceutical Sciences, University of California, Irvine, Irvine, California 92697, United States
[4]Sue and Bill Gross Stem Cell Research Center, University of California, Irvine, Irvine, California 92697 United States
[5]Department of Chemistry, University of California, Irvine, Irvine, California 92697, United States

[*]Corresponding authors: aluptak@uci.edu (A.L.)

Supplementary Information

DNA and RNA controls

Human RNase P (RPP30) was amplified from the Hs_RPP30_Positive control plasmid (Integrated DNA Technologies, IDT, 10006626). SARS-CoV-2 N1 DNA was amplified from the 2019-nCoV_N_Positive control plasmid (IDT, 10006625). SARS-CoV-2 viral particles and synthetic RNA were provided by the XPRIZE Foundation (Team ID: 3650) and produced by ZeptoMetrix and Twist Biosciences, respectively. Viral particles were stored at 4 °C per manufacturer recommendation. Synthetic RNA was stored at -80 °C per manufacturer recommendation.

RT-qPCR primer design

Human RNase P (RPP30) primers and probe were synthesized by IDT (oligonucleotides: 25 nmol, standard desalting; hydrolysis probe: 25 nmol, HPLC purified). SARS-CoV-2 N1 and N2 primers and probes were synthesized by IDT (oligonucleotides: 25 nmol, standard desalting; hydrolysis probe: 25 nmol, HPLC purified). Beta-actin (ACTB) primers and probe (IDT, Hs.PT.39a.22214847) were synthesized by IDT (oligonucleotides: 25 nmol, standard desalting; hydrolysis probe: 25 nmol, HPLC purified). Oligonucleotides and probes were resuspended in 10 mM Tris-HCl, pH 7.5 and 0.1 mM EDTA to 100 µM. RNase P and SARS-CoV-2 N1 primers and probes were designed for research-use only by the Centers for Disease Control and Prevention[1]. Primer and probe sequences are listed in Table S1.

RT-LAMP primer design

N1 RT-LAMP primers were designed by Huang *et al.* for the SARS-CoV-2 *N* gene[1]. Oligos were synthesized by IDT (25 nmol, standard desalting). Oligomers were resuspended in 10 mM Tris-HCl, pH 7.5 and 0.1 mM EDTA to 100 µM. RT-LAMP sequences can be found in Table S1.

Test	Target	Name	Sequence
RT-qPCR	Human RNase P[1] (RPP30; NM_006413.5)	RNase P Forward	AGATTTGGACCTGCGAGCG
		RNase P Reverse	GAGCGGCTGTCTCCACAAGT
		RNase P Probe	FAM-TTCTGACCTGAAGGCTCTGCGCG-BHQ1
RT-qPCR	SARS-CoV-2 N1[1] (N; NC_045512.2)	2019-nCoV_N1 Forward	GACCCCAAAATCAGCGAAAT
		2019-nCoV_N1 Reverse	TCTGGTTACTGCCAGTTGAATCTG
		2019-nCoV_N1 Probe	FAM-ACCCCGCATTACGTTTGGTGGACC-BHQ1
RT-qPCR	SARS-CoV-2 N2	N2 Forward	TTACAAACATTGGCCGCAA
		N2 Reverse	GTGACTTCCATGCCAATGC
		N2 Probe	SUN-ACAATTTGCCCCCAGCGCTTCAG-3IABkFQ
RT-qPCR	Human beta-actin (ACTB; NM_001101.5)	ACTB Forward	TAACAGATTGATGATGCATGAAATGGG
		ACTB Reverse	CCCATGAGTGGCTCCTAAAGCAGCTGC
		ACTB Probe	(see IDT: Hs.PT.39a.22214847)
RT-LAMP	SARS-CoV-2 N1[2]	F3	TGGACCCCAAAATCAGCG
		B3	GCCTTGTCCTCGAGGGAAT
		FIP	CCACTGCGTTCTCCATTCTGGTAAATGCACCCCGCATTACG
		BIP	CGCGATCAAAACAACGTCGGCCCTTGCCATGTTGAGTGAGA
		LF	TGAATCTGAGGGTCCACCAAA
		LB	GGTTTACCCAATAATACTGCGTCTT

Supplementary Table 1: RT-qPCR and RT-LAMP primers. Human RNase P and SARS-CoV-2 primers and probes were designed by the Centers for Disease Control and Prevention[1]. SARS-CoV-2 N2 primers and probe were designed for this study. Human beta-actin (ACTB) primers and probe were designed by IDT (Hs.PT.39a.22214847). SARS-CoV-2 N1 RT-LAMP primers were designed by Huang *et* al[2]. All primers were purchased and synthesized by IDT.

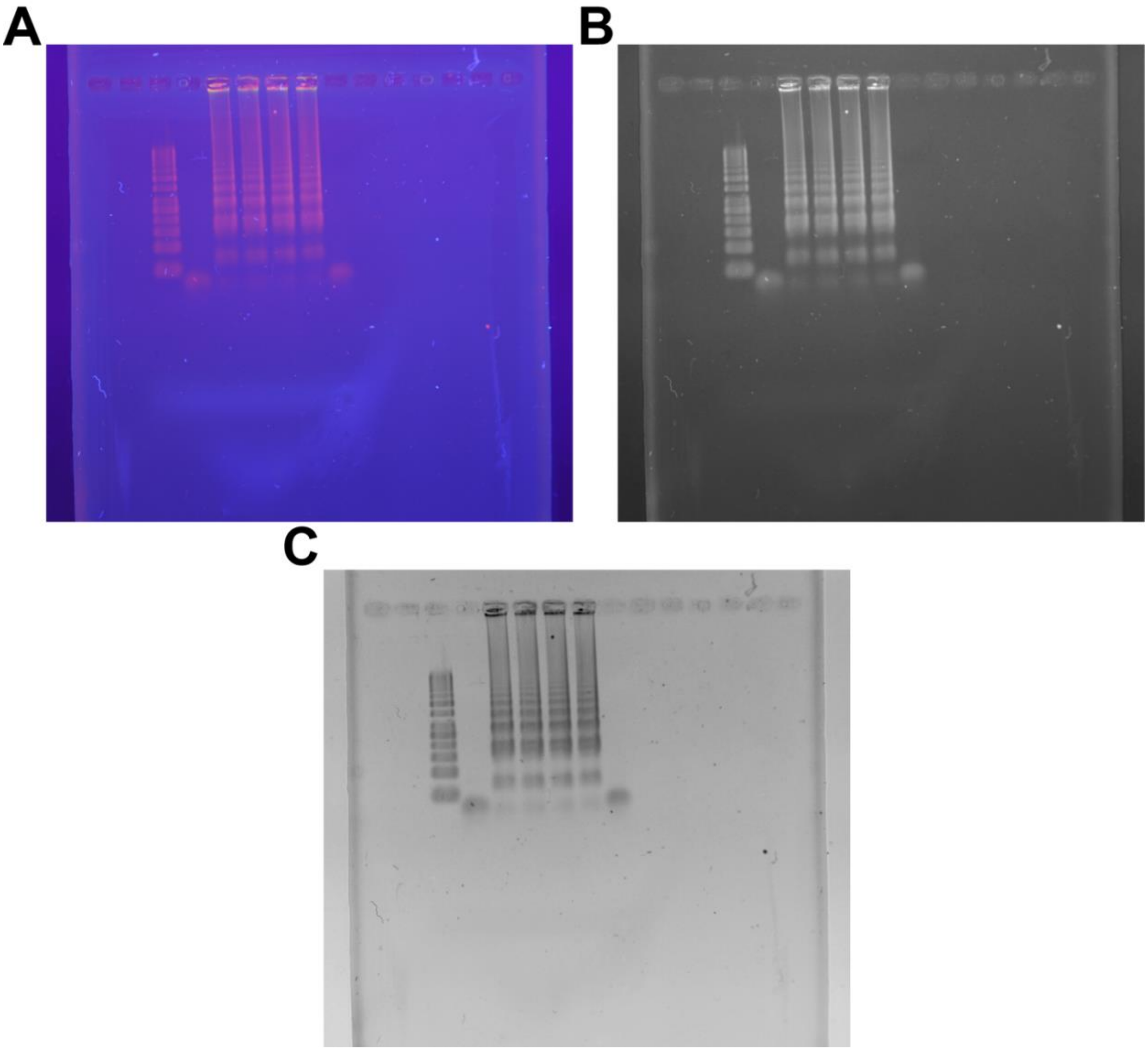

Supplementary Figure 1: Full-length agarose gel from Figure 5B. (**A**) Original uncropped gel image. (**B**) Red-channel image of gel for ethidium bromide visualization and contrast. (**C**) Inverted red-channel image.

References

1 Centers for Disease Control and Prevention. *Real-time RT-PCR Primers and Probes for COVID-19*, https://www.cdc.gov/coronavirus/2019-ncov/lab/rt-pcr-panel-primer-probes (2020).

2 Huang, W. E. *et al.* RT-LAMP for rapid diagnosis of coronavirus SARS-CoV-2. *Microb Biotechnol* **13**, 950-961, doi:10.1111/1751-7915.13586 (2020).
